# On The Role of Natural Killer Cells in Neurodegenerative Diseases

**DOI:** 10.3390/toxins5020363

**Published:** 2013-02-19

**Authors:** Azzam A. Maghazachi

**Affiliations:** Department of Physiology, Institute of Basic Medical, University of Oslo, Oslo 0317, Norway; E-Mail: azzam.maghazachi@medisin.uio.no; Tel.: +47-22851203; Fax: +47-22851279

**Keywords:** NK cells, neurodegenerative diseases, multiple sclerosis, globoid cell leukodystrophy

## Abstract

Natural killer (NK) cells exert important immunoregulatory functions by releasing several inflammatory molecules, such as IFN-γ and members of chemokines, which include CCL3/MIP-1α and CCL4/MIP-1β. These cells also express heptahelical receptors, which are coupled to heterotrimeric G proteins that guide them into inflamed and injured tissues. NK cells have been shown to recognize and destroy transformed cells and virally-infected cells, but their roles in neurodegenerative diseases have not been examined in detail. In this review, I will summarize the effects of NK cells in two neurodegenerative diseases, namely multiple sclerosis and globoid cell leukodystrophy. It is hoped that the knowledge obtained from these diseases may facilitate building rational protocols for treating these and other neurodegenerative or autoimmune diseases using NK cells and drugs that activate them as therapeutic tools.

## 1. Introduction

The immune system is composed of a network of cells and proteins that act in concert to defend the body against diseases. There is increasing evidence that key components of the innate immune system, such as natural killer (NK) cells, are instrumental in disease control or progression. These cells have both cytolytic and immunoregulatory functions [1] and play vital roles by surveilling the microenvironment searching for abnormal growth or microbial-infected cells [2]. NK cells recognize and kill these latter cells by a set of receptors known as NK cell cytotoxicity receptors [3]. Therefore, NK cells are important in protection, as well as therapy against cancer [4,5], in lysing virally-infected cells [6] and are implicated in therapy against HIV-infection [7]. More than 90% of NK cells are found in the blood circulation, whereas less than 10% are found in tissues, such as spleen, liver and lungs [8]. NK cells are not circulatory [9]; however, under pathological conditions and during inflammation, they extravasate into the lymph nodes and accumulate at sites of tumor growth [10]. In addition, NK cells with phenotypes and functional activities similar to activated NK cells can be found in the liver of normal rats [11]. These observations suggest that NK cells found in secondary lymphoid tissues or in non-lymphoid tissues have been stimulated *in situ* before their extravasation into these areas. It is not clear why the majority of NK cells are found in the circulation and what influence their accumulation in peripheral tissues. The majority of peripheral blood resting NK cells (about 80%–90%) do not express the CD56 molecule, and are designated as CD56^dim/−^ cells. These cells have high cytolytic activity and low immunoregulatory capacity, whereas the other 10%–20%, designated as CD56^bright/+^ cells, have high immunoregulatory function and low cytolytic activity [8,12]. In addition to their role in cancer [4,5], NK cells are involved in autoimmune diseases, such as multiple sclerosis [13], systemic lupus erythematosus [14] and rheumatoid arthritis [15]. The model described by others [16], is perhaps doubtful to have any significant impact for human diseases, and will not be further discussed.

### 1.1. Natural Killer (NK) Cells Express G Protein-Coupled Receptors

NK cells express receptors, which bind members of the heptahelical receptors (also known as G protein-coupled receptors, “GPRC”, or seven transmembrane spanning domain receptors). Both CD56^bright/+^ and CD56^dim/−^ NK cells express the sphingosine 1-phosphate receptors S1P_1_, S1P_4_ and S1P_5_ [17]. In addition, both subsets express LPA_1_, LPA_2_ and LPA_2_, the receptors for lysophosphatidic acid [18]. The expression of lysolipid receptors on the surface of these cells may facilitate their migration into various tissues, since lysophospholipids are secreted by tumor and inflammatory cells [19]. Although NK cells have been implicated in several neurodegenerative diseases, such as Alzheimer’s disease [20,21], this review will focus on two neurodegenerative diseases, where the author developed experience with, namely multiple sclerosis and globoid cell leukodystrophy. 

### 1.2. NK Cells Contain Different Subsets

**Figure 1:**
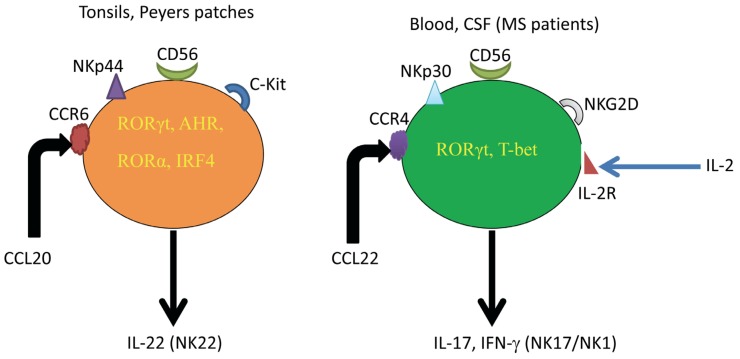
Comparison among NK22 cells and NK17/NK1 cells. While NK22 cells are found in tissues, such as tonsils and peyers patches, NK17/NK1 cells are generated from human blood after activation *in vitro* with IL-2 and are abundant in the CSF of MS patients without activation. NK22 cells express the NK cytotoxicity receptor NKp44, whereas NK17/NK1 cells express NKp30, NKG2D, as well as NKp44, NKp46 and CD158, but to a lower extent than the other NK cell cytotoxicity receptors. Whereas NK22 cells express CCR6, secrete and respond CCL20/MIP-3α, NK17/NK1 cells express CCR4, secrete and respond to CCL22/MDC. As for the transcription factors, NK22 cells express RORγ, AHR, RORα and IRF4, while NK17/NK1 cells express RORγ and T-bet and, consequently, secrete both IL-17 and IFN-γ.

Previously, human NK cells have been classified based on the expression of the CD56 molecule, but other findings suggest that NK cells might be classified into NK1 cells producing Th1-like cytokines, such as IFN-γ, and NK2 cells producing Th2-like cytokines, such as IL-5 and IL-13 [22]. Human NK cells are also divided into cells that express the chemokine receptors, CXCR1, CXCR3 and CXCR4, while others express CCR1, CCR4, CCR5, CCR6, CCR7, CCR9, CXCR5 and CXCR6 within both CD56^bright^ and CD56^dim^ subsets [23]. Another set of NK cells isolated from human peyers patches or tonsils expressing NKp44 and CCR6 molecules and secreting IL-22 and CCL20/MIP-3α/LARC was discovered and the cells were designated as NK22 [24]. Yet, other cells isolated from intestinal lamina propria, expressing NKp46^+^ NKG2D^+^ NK1.1^int^ RORγ^high^ and secreting IL-22, have been described [25]. Human NK cells in stage III development express CD34^+^CD117^+^2B4^+^ that produced IL-22 and IL-26 but not IL-17, have been isolated from tonsil tissues [26]. Further work from our group described a new subset of human NK cells isolated from the peripheral blood and activated *in vitro* with IL-2. The cells secrete IL-17 and IFN-γ and were designated as NK17/NK1 cells [27]. Figure 1 shows the phenotypic expression of NK22 cells and NK17/NK1 cells.

Although it is well established that IFN-γ is an inflammatory cytokine, the function of IL-17 in autoimmune diseases has not been clarified. IL-17 is considered an inflammatory molecule, because it induces the production of IL-6, TNF-α or CXCL8/IL-8 [28], stimulates the production of matrix metalloproteinases-2, 3, 9 and 13 [29], and facilitates the proliferation of endothelial cells [30], but it may relieve inflammatory colitis disease [31]. In addition, it was recently reported that this cytokine may be beneficial in myocardial infarction, due to its ability to inhibit the chemotaxis of monocytes in this disease [32]. We also reported that IL-17 tends to reduce the expression of two major chemokine receptors involved in monocyte migration into inflamed hearts, namely CCR2 and CXCR4 [32]. 

## 2. Role of NK Cells in Multiple Sclerosis (MS)

Multiple sclerosis (MS) is an inflammatory disorder that results in demyelination and destructions of neurons. The animal experimental model of MS, also known as experimental autoimmune encephalomyelitis (EAE), is an excellent model, which describes the role that autoreactive CD4^+^ cells play in this disease [33,34]. A hallmark of the immune response in MS is the formation of isolated areas of inflammation called MS lesions. Lesions can appear both in the white matter and in the grey matter of the brain and are often found around the ventricles, in the optic nerve, in the brain stem and in the spinal cord [35]. 

The role of NK cells in MS is controversial, with one school of thought suggests that NK cells ameliorate the disease, whereas another school indicates that they exacerbate the disease [36,37]. The first indicates that NK cells ameliorate EAE by lysing cells that aggravate the MS disease [38]. Consequently, depleting NK cells before immunization of sensitive mice with myelin oligodendrocyte glycoprotein (MOG35–55) peptide leads to a severe relapsing EAE, which is the result of increased T-cell proliferation and production of Th1 cytokines. NK cells, by suppressing pathogenic autoreactive T-cells, which mediate the central nervous system (CNS) inflammation, may repair the demyelination [39]. Impaired NK cell recruitment into CX_3_CR1-deficient mice suffering from EAE also leads to severe disease [40]. This is because recruitment of NK cells by CX_3_CR1/Fractalkine axis into the CNS of EAE mice inhibits the inflammatory Th17 cells [41], and hence, lack of NK cells may result in enhancing the severity of the disease.

In contrast, others suggested that NK cells exacerbate MS/EAE. For example, higher NK cell activity is correlated with a higher risk of developing active lesions in relapsing-remitting MS patients [42]. Also, IL-12 produced by astrocytes promotes NK cell developments that secrete cytokines, which enhance T-cell activation [43]. Further, IL-18 produced during the primary injection of antigens increased IFN-γ secretion by NK cells. IFN-γ in turn activates autoreactive Th1 responses, whereas an impaired capacity of NK cells to release IFN-γ is a major mechanism underlying resistance to EAE [44]. In these studies, it was observed that depleting NK cells with specific antibodies leads to diminished EAE clinical disease [45]. 

**Figure 2:**
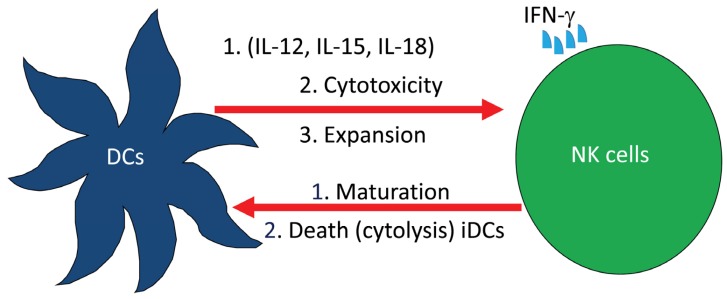
Complex interactions among cells of the innate immune system. Dendritic cells (DCs) releasing IL-12, IL-15 and IL-18 activate natural killer (NK) cell cytotoxicity, expansion and the release of IFN-γ. In turn, NK cells induce either the maturation or death of DCs.

We examined the role of NK cells in both EAE experimental system and MS patients. Our approach was to investigate whether drugs used to treat MS patients may exert effects on NK cell biological activities. One of the drugs we used is glatiramer acetate (GA; commercial name Copaxone), which is made up of four amino acids, namely Glu, Ala, Lys and Tyr, that are found in myelin. This drug prevents the incidence of experimental autoimmune encephalomyelitis in animals and reduces relapses in patients with MS [46]. MS patients receiving GA had a steady decline in disease relapses with neurological improvement when compared to patients receiving placebo [47,48]. We recently described the activities of NK cells and dendritic cells (DCs) isolated from nine relapsing remitting MS patients. Our results demonstrate that NK cells generated from the peripheral blood of these patients have increased cytolytic activity against tumor target cells, autologous immature (i) and mature (m) DCs [49]. NK cells are known to interact with DCs leading to DCs maturation or death and/or NK cells activation [50]. It was also suggested that the interaction between NK cells and DCs is bidirectional and involves cell-to-cell contact, where DCs activate NK cells by enhancing their proliferation, cytotoxic activity and IFN-γ production, whereas activated NK cells provide either maturation signals for DCs or induce their death by direct killing (Figure 2). 

It is not yet known where in the body NK cells interact with DCs, however, there is a consensus suggesting that this interaction might take place at inflammatory sites [50]. Our results showing that NK cells lyse DCs after treatment may provide a mechanistic explanation for such an interaction, leading to a vitally important sequence of events, which culminate in benefiting MS patients. In addition, the observations provide a novel mechanism of action for drugs used to treat autoimmune diseases, showing that these drugs ablate DCs by activating NK cells, which lyse them. 

*In vitro* work also revealed that GA enhances the cytolysis of activated human NK cells against immature and mature monocyte-derived DCs [51]. These findings were supported in mice with EAE, where administration of GA ameliorated the EAE clinical scores, corroborated with the ability of NK cells to kill both immature and mature DCs [52]. Collectively, these findings demonstrate that GA inhibition of EAE clinical disease may be due to its ability to activate NK cells to lyse DCs, among other functions. The finding that NK cells exposed to GA kill both immature DCs and mature DCs leads to the inability of presenting antigens to autoreactive T-cells. Finally, we suggest that the newly described NK17/NK1 cells may be of interest in MS disease. We are currently evaluating the effects of various drugs used to treat MS patients on the biological activities of these cells. Also, we are examining whether these cells may secrete anti-inflammatory cytokines, such as IL-10 among others, notwithstanding the perplexing and highly questionable results, showing that IL-10 is a culprit in another autoimmune disease [53]. 

## 3. Role of NK Cells in Globoid Cell Leukodystrophy (GLD)

**Figure 3:**
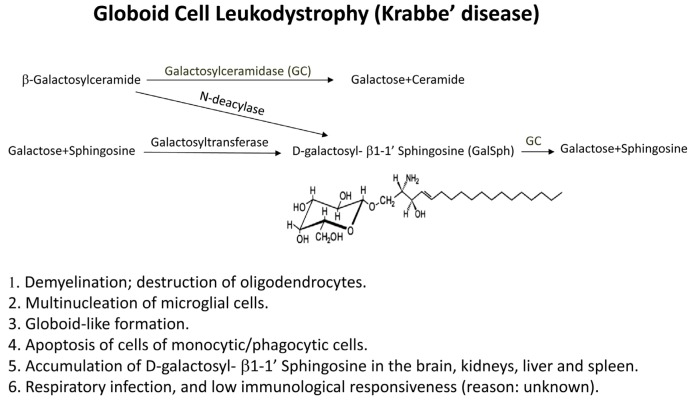
Formation of glycosphingolipids. Galactosylceramidase (GALC or GC) is deficient in patients with globoid cell leukodystrophy (GLD) or in twitcher mice, resulting in the accumulation of the toxic lipid (GalSph), which, under normal conditions, is converted into galactose and sphingosine by GC. Some characteristics of GLD disease are described in [1,2,3,4,5,6].

Globoid cell leukodystrophy (GLD), or Krabbe disease, is an autosomal recessive disease that affects infants [54,55,56]. Patients who are homozygous for this disease are presented between 3 and 6 months of age with irritability, spasticity and mental deterioration. These patients died before the age of two years from hyperpyrexia or respiratory infections. The pathology of GLD is characterized by the destruction of oligodendrocytes, reduced myelin formation and the accumulation of globoid cells, not only in humans, but also in canines, domestic cats and rhesus monkeys [57,58,59,60]. Accumulation of the toxic lipid D-galactosyl-β1-1’ sphingosine (galactosylsphingosine = GalSph) in the brain is thought to cause the disease [55,61,62]. Galactosylsphingosine is virtually absent from normal brain or other tissues [63], but it accumulates at high concentrations in the brain of Krabbe patients, due to the deficiency of the enzyme galactosyl ceramidase, “GALC or GC” (Figure 3). 

Twitcher mice, which have mutations in their GALC gene, represent an authentic model of human Krabbe disease [64]. Galactosylsphingosine is increased in the kidneys, liver, spleen and highly accumulates in the brain of these animals. For example, the level of this lipid can reach up to 15 nM/g body weights in the brain of twitcher mice and about 60 ng/100 mg weights in the spleen of these mice [61]. Galactosylsphingosine induces the *in vitro* apoptosis of rat C6 glial cells [65] or the oligodendrocytes MO3.13 cell line [66]. Apoptotic cells were also observed in the brain of patients with various lysosomal storage diseases, such as Tay-Sachs and Sandhoff diseases, as well as the brain of mice with GM2 gangliosidosis [67]. A heptahelical receptor that binds heterotrimeric G proteins, named T-cell-death-associated gene 8 “TDAG8” [68,69], has been shown to bind GalSph, as well as glucosylated sphingosine “GlcSph”. Human TDAG8 exists in normal tissues and is restricted to lymphoid organs, such as spleen and lymph nodes, as well as its expression in peripheral blood lymphocytes. We reported that human NK cells express TDAG8 and demonstrated that GalSph and GlcSph damage these cells [70]. Although GalSph and GlcSph induce the apoptosis and multinucleation in NK cells, most other lipids examined did not affect the morphology or induced apoptosis of these cells [70]. 

Patients with storage diseases that involve the nervous system succumb to infections, which are the major cause of death in these patients [71]. The reason for increased rate of infections is not clear. Our results showing that galactosylsphingosine induces the *in vitro* chemotaxis of NK cells [70] and their accumulation in twitcher mice splenic white pulps [52], corroborated with induction of their apoptosis, may provide an explanation to this enigma. In fact, it was very difficult to isolate NK cells from the spleens of twitcher mice in order to examine their *in vitro* activities, which could be due to their apoptosis [52]. In addition to NK cells, cells with Ia^+^ phenotype that include macrophages and dendritic cells also disappeared from the spleens of twitcher mice. These findings may provide an explanation to the observations that twitcher mice have low immunological activity and can easily accept grafts [72]. In line with these observations is the demonstration that macrophages may counteract the demyelination in twitcher mice [73]. 

Transplantation of hematopoietic cells from normal mice into syngeneic twitcher mice resulted in increased survival, but in these early studies, the success of the transplantation procedures did not go beyond 100 days [74,75]. However, recent advancements revealed that administration of murine bone marrow-derived stem cells via intraperitoneal injection resulted in improved lifespan and reduced twitching severity and frequency [76]. In humans, Krivit *et al*. [77] reported a highly successful rate of recovery in five patients with late-onset GLD after hematopoietic stem cell transplantation, and the success of these transplantation procedures was attributed to the ability of donor macrophages to provide the enzyme necessary to hydrolyze galactosylsphingosine. Based on these observations, it was shown that administration of the enzyme GALC inserted into adeno-virus to twitcher mice resulted in the accumulation of this enzyme in the brain of these mice, which consequently led to prolong their lives [78]. Further, recent studies demonstrated that a combination of enzyme replacement therapy with bone marrow transplantation may have a great promise for treating twitcher mice [79,80]. It is not clear whether NK cells were involved in these therapeutic regiments. However, if indeed NK cells were present in the grafts, then these cells may have helped in resolving the infections associated with the disease. 

## 4. Conclusions

Although two concepts were put forward in the literature regarding the role of NK cells in MS disease, a plausible explanation for these contradictory effects is the presence of different subsets of NK cells. It is clear that the beneficial effects of drugs used to treat MS are always associated with increased NK cytolytic activity against target cells. Therefore, it can be concluded that activation of NK cells in MS patients may be an important factor for subsiding the severity of the disease. The discovery of NK17/NK1 cells should add another important value of using these cells in treating autoimmune diseases and, in particular, MS. 

Regarding another neurodegenerative disease, NK cells express TDAG8, the receptor for glycosphingolipids galactosyl sphingosine (GalSph) or glucosyl sphingosine (GluSph) and that GalSph and GluSph damage NK cells by inducing their apoptosis. In addition, GalSph induces human NK cell chemotaxis *in vitro* and promotes the localization of mouse NK cells in the white pulp areas of twitcher mice spleens, corroborated with the induction of their apoptosis. Whether these lipids perform similar functions in the brain of patients suffering from the disease is an issue that has not yet been investigated. The involvement of NK cells may provide an explanation to the observation that patients with lysosomal storage diseases die of respiratory infections as their NK cells are destroyed by the glycosphingolipids. Collectively, these observations should provide new insights into neurodegenerative diseases, and the knowledge can be utilized for formulating new strategies to treat these diseases.
